# Influence of leg axis alignment on MRI T2* mapping of the knee in young professional soccer players

**DOI:** 10.1186/s12891-024-07233-3

**Published:** 2024-02-15

**Authors:** D. Dalos, P. R. Marshall, M. Lissy, K. J. Maas, F. O. Henes, M. G. Kaul, H. Kleinertz, J. Frings, M. Krause, K. H. Frosch, G. H. Welsch

**Affiliations:** 1 Center for Athletic Medicine, UKE Athleticum, University Medical Center Hamburg-Eppendorf, Hamburg, Germany; 2 Institute of Interdisciplinary Exercise Science and Sports Medicine, MSH Medical School Hamburg, Hamburg, Germany; 3 Department of Trauma and Orthopaedic Surgery, University Medical Center Hamburg-Eppendorf, Hamburg, Germany; 4 RasenBallsport Leipzig GmbH, Leipzig, Germany; 5 Department of Orthopaedic and Trauma Surgery, Martin-Luther-University Halle-Wittenberg, Halle, Germany; 6 Department of Diagnostic and Interventional Radiology and Nuclear Medicine, University Medical Center Hamburg-Eppendorf, Hamburg, Germany; 7 Department of Trauma Surgery, Orthopaedics and Sports Traumatology, BG Hospital Hamburg, Hamburg, Germany; 8 Department of Diagnostic and Interventional Radiology, BG Hospital Hamburg, Hamburg, Germany

**Keywords:** Leg axis, Knee MRI, T2* mapping, Professional soccer, Varus alignment

## Abstract

**Background:**

Investigation of the association between leg axis alignment and biochemical MRI in young professional soccer players in order to identify a potential influence of the leg axis on cartilage regions at risk.

**Methods:**

Sixteen professional soccer players (21 ± 3 years) underwent static and dynamic leg axis analysis via radiation free DIERS formetric 4 D as well as 3-T MRI examination of both knees. Quantitative T2* mapping of the knee cartilage was performed and T2* values were evaluated as 144 regions of interest. Subgroup analysis was performed in players with severe varus alignment (> 6°).

**Results:**

Analysis of the leg axis geometry revealed a mean static alignment of 6.6° ± 2.5 varus and a mean dynamic alignment of 5.1° ± 2.6 varus. Quantitative T2* mapping showed significantly increased T2* values in the superficial cartilage layer compared to the deeper region (*p* < 0.001) as well as a significant increase in relaxation times in the femoral cartilage from anterior to intermediate to posterior (*p* < 0.001). Combination of both methods revealed a significant correlation for the degree of varus alignment and the femoral, posterior, deep region of the medial knee compartment (*r* = 0.4; *p* = 0.03). If severe varus alignment was present this region showed a significant increase in relaxation time compared to players with a less pronounced leg axis deviation (*p* = 0.003).

**Conclusion:**

This study demonstrates that varus alignment in young soccer players is associated with elevated T2* relaxation times in the deep cartilage layer of the medial, posterior, femoral compartment and might therefore be a contributing factor in the early pathogenesis of manifest cartilage lesions. Therefore, these findings should be considered in the development of preventive training programs.

## Introduction

Professional soccer players demonstrate an increased prevalence of cartilage alterations and cartilage lesions of the knee [1, 2]. Its potential progression to osteoarthritis is an important complication in former elite athletes [3, 4]. Several studies document the influence of the leg axis on the development of these lesions. Varus alignment of the knee can increasingly be found in professional soccer players and is discussed to induce cartilage lesions of the medial compartment with a potential progression to end-stage varus osteoarthritis [5–10]. Cartilage lesions of the medial compartment of the knee are described to be present in up to 85% of long-term professional soccer players [2].

Determination of the leg axis is usually performed by weight bearing full-length radiograph of the lower limbs, but the inevitable radiation exposure does not make this method feasible, especially in asymptomatic patients and underage players. Radiation-free measurement methods of the leg axis alignment include the caliper method, long-arm goniometer as well as newer gait analysis systems allowing not only evaluation of static but of dynamic limb angles and loads as well [11–14]. Recently Ossendorf et al. reported a high accuracy for a two-dimensional motion capture analysis system by demonstrating strong correlations between full-length radiography and said system under static and dynamic conditions [15].

In order to identify cartilage lesions at an early stage, quantitative biochemical MRI diagnostics have emerged as a viable option in recent years. The changes of internal organization of collagen fibres and associated change in water retention can lead to an increase of T2* relaxation times. This analysis of relaxation times of the articular cartilage can detect changes before morphological visualization is possible [16, 17]. Especially in professional soccer players early knowledge of potential cartilage lesions and its association to the leg axis is of particular preventative importance. Up to date there is no study examining the influence of static and dynamic leg axis on T2* measurements of the knee cartilage. Therefore, the purpose of this study is to analyze the association between biochemical MRI and leg axis analysis in young professional soccer players in order to identify the potential influence of the leg axis on early cartilage regions at risk. The hypothesis of the study is that the degree of varus deformity effects T2* relaxation times of the medial joint compartment.

## Methods

This study was approved by the local ethics committee (Ethical Review Committee Hamburg, Germany; study number: PV5923) and conducted in accordance with the ethical standards laid out by the 1964 Declaration of Helsinki.

### Study population

The study population included the analysis of a youth male soccer team of a German professional soccer club. All participants have passed a professional soccer training in a professional club youth academy for at least four years. Exclusion criteria included previous relevant knee injury, prior knee surgery and current injuries or pain resulting in actual down time. Overall, 16 participants with a mean age of 21 ± 3 years met the inclusion criteria and could be recruited. MRI and leg axis analysis were carried out in each player for each leg on the same day around midday. Analysis was performed by one experienced sports medicine physician specializing in movement analysis (leg axis analysis) and in consensus by two radiologists with three and twelve years of experience, respectively (MRI T2* mapping). Due to technical error one leg of one participant was not examined, resulting in a total of 31 legs available for analysis.

### MRI acquisition and image analysis

MRI examination was performed using a 3 T MR system (Ingenia, Philips, Best, The Netherlands) as previously described [18–20]. Prior to the MRI examination all participants kept a resting period of 30 min in the supine position to reduce any possible influence of daily physical loading on quantitative measurements. As the signal receiver, a dedicated 16-channel knee coil was used with maximum knee flexion of 10 degrees. The imaging protocol consisted of a 3D PDw Fast Spin Echo (FSE) sequence with multiplanar reformations as well as a T1w FSE sequence in coronal orientation. For quantitative analysis, a sagittal 3D T2* weighted Gradient Echo (GRE) sequence was applied with 22 echo times (TE: 4.6 – 53.6).

T2* mapping was performed as previously described for each knee of the participants [18–20]. All MRI data sets were analyzed in a radiology department using a commercially available post processing workstation (Extended Brilliance Workspace, Version 2.0, Philips Healthcare, Best, The Netherlands). The cartilage regions of interest (ROI) were defined within the weightbearing part of the femorotibial joint and drawn as follows: Segmentation of the cartilage thickness in a superficial (cartilage surface to the middle of the cartilage) and deep layer (from the middle of the cartilage to the osteochondral interface). Segmentation into an anterior, intermediate and posterior zone (bordered by the peripheral margins of the menisci in the sagittal plane). Segmentation according to the joint compartments in medial and lateral with an additional division in the frontal plane into segments 1–3 (lateral slices) and 4–6 (medial slices). Overall, 144 different ROIs could be defined in each knee and represent the weightbearing cartilage of the femorotibial joint.

### Leg axis analysis

**Fig. 1:**
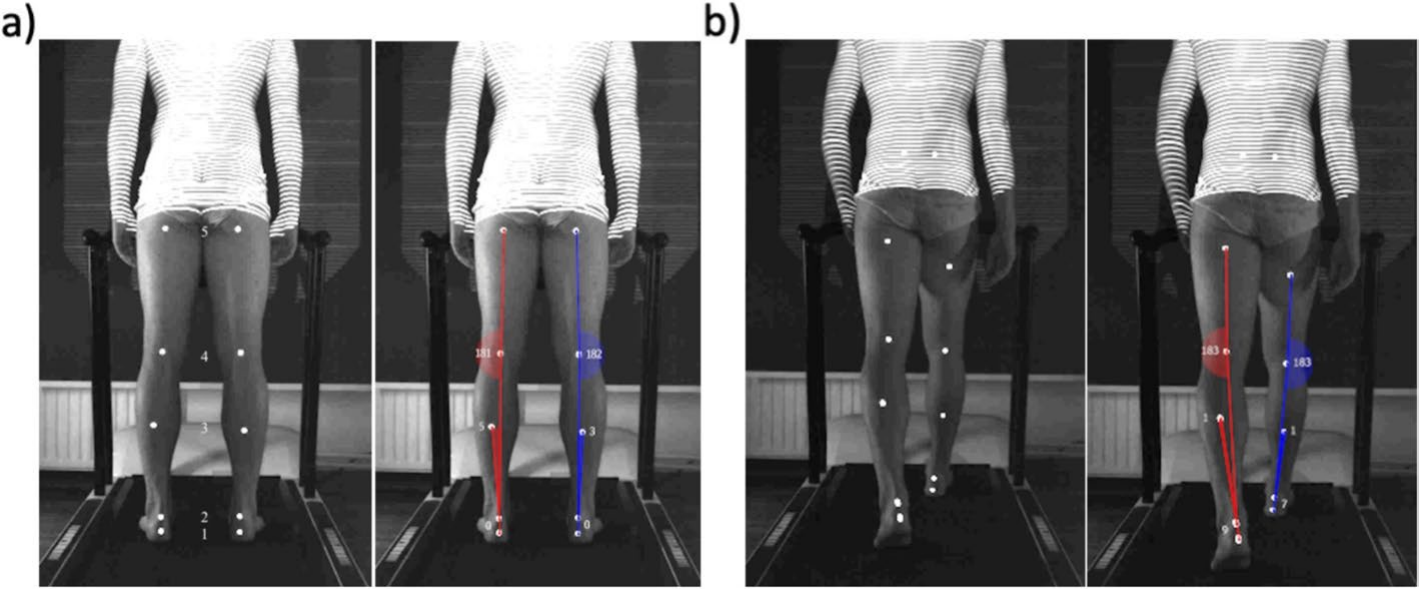
Exemplary measurement of static and dynamic leg axis alignment via DIERS formetric 4D System. **a** Static measurement: Marker placement on defined landmarks (soft spot directly below (1) and above (2) the calcaneus, insertion point of the medial and lateral head of the gastrocnemius muscle into the tendon (3), center of the popliteal fossa (4), center of the upper thigh directly beneath the gluteal fold (5)) and determination of static knee angle. Further leg angles indicated were not utilized in this study. **b** Dynamic measurement: Marker placement and determination of dynamic knee angle were performed as described in **a**

Determination of the leg axis was performed as previously described using the DIERS formetric 4D (DIERS international, Schlangenbad, Germany) motion analysis system for each leg of the participants [15]. This system provides two-dimensional video documentation of leg axis geometry in the frontal plane from a posterior view under static and dynamic conditions. Its accuracy for static as well as dynamic measurements was recently validated by Ossendorf et al. in comparison to long-standing-leg radiography [15]. According to the manufacturers´ instructions five tracking skin markers were placed on defined anatomic landmarks of each leg. Defined landmarks include the soft spot directly below (1) and above (2) the calcaneus, the insertion point of the medial and lateral head of the gastrocnemius muscle into the tendon (3), the center of the popliteal fossa (4) of the knee and the center of the upper thigh directly beneath the gluteal fold (5) (Fig. 1). During analysis the reflecting skin markers are spotlighted and automatically registered through the recording station (DIERS leg axis accessory of DIERS formetric 4D, DIERS international, Schlangenbad, Germany). The station is positioned centered and orthogonally behind the treadmill allowing accurate measurement in the frontal plane. The correct positioning of each participant was carefully checked and assured with the feet placed on parallel lines on the treadmill. The skin markers divide each leg into smaller segments allowing for automatic calculation of the mechanical leg axis which is reflected by the connective lines between the markers 2, 4 and 5 (Fig. 1). Data recording, processing, and export were performed with a system-exclusive software (DICAM 3, DIERS International GmbH, Schlangenbad, Germany) [15]. Knee angles of < 180° (negative values) indicate valgus alignment and angles of > 180° indicate varus alignment (positive values) [15, 21]. Static measurements were carried out with the patient standing in upright, neutral position, full weight bearing and barefooted, wearing only underwear (Fig. 1a). Dynamic measurements were carried out in a similar manner but on the treadmill at 5 km/h for overall 5 steps (Fig. 1b).

### Data analysis

Mean values and confidence intervals were ascertained for all of the defined 144 regions of interest of MRI T2* values. Mean values and standard deviation were ascertained as well for static and dynamic leg axis measurements. To assess the mean dynamic value of all players, the mean value of all five steps for each player was calculated beforehand.

Furthermore, to analyze the effect of varus deformity a subgroup analysis was performed comparing T2* values of each ROI of players with a static varus alignment > 6° (*n* = 10, 20 legs) to the other players of the study cohort.

Statistical analysis was performed with analysis of variance using SPSS (IBM SPSS 23.0 IBM Corp., Armonk, NY, USA) with a significance level of α = 0.05. Analysis was carried out for leg side (entire left vs right knee), cartilage layers (entire superficial vs deep), joint compartments (entire medial vs lateral) as well as zones (entire anterior vs intermediate vs posterior). Potential correlation between leg axis and MRI T2* values was displayed by calculating the Pearson correlation coefficient through linear regression analysis for each cartilage region.

## Results

### MRI T2* mapping

**Fig. 2:**
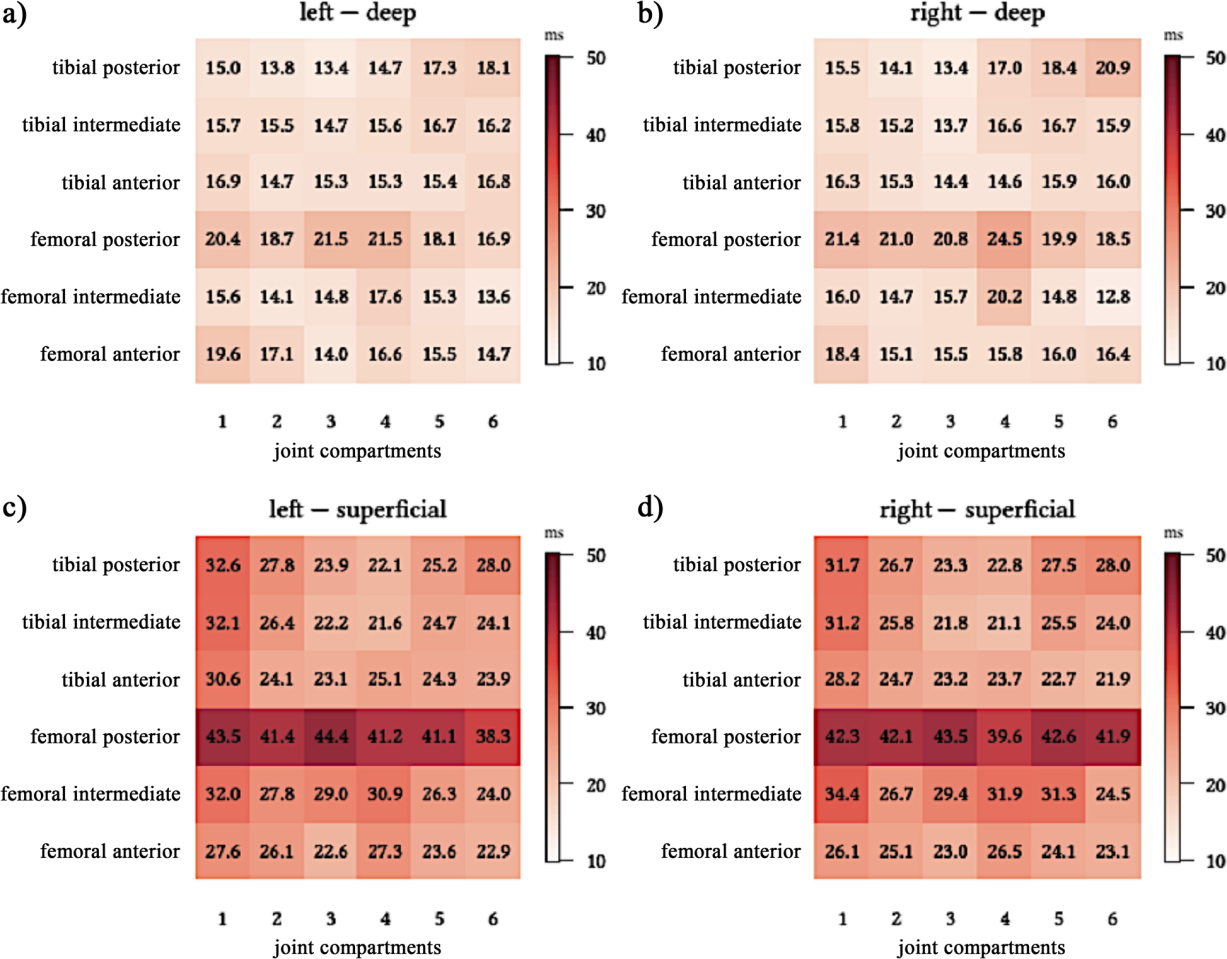
Mean values of T2* relaxation times in ms of all 144 cartilage regions of interest of the femorotibial joint. In each figure section the numbers 1 to 6 (x-axis) represent the segmentation in the frontal plane from lateral to medial (1-3: lateral joint compartment; 4-6: medial joint compartment). The segmentation of the cartilage in the sagittal plane is defined as anterior, intermediate and posterior (y-axis). The regions of interest are shown for **a**) the deep cartilage layer of the left knee **b**) the deep cartilage layer of the right knee **c**) the superficial cartilage layer of the left knee **d**) the superficial cartilage layer of the right knee

**Fig. 3:**
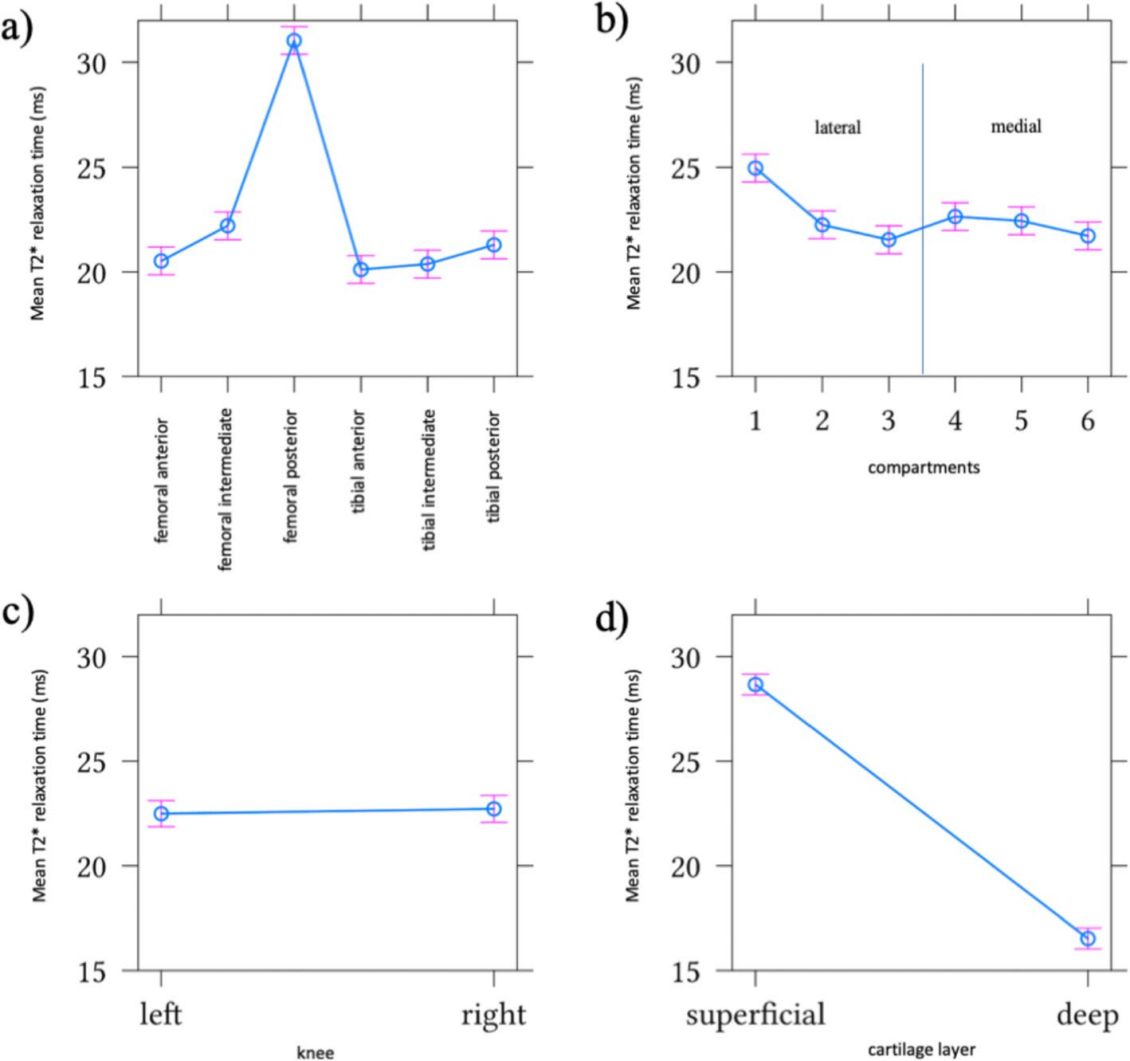
Effect of joint zones (**a**), joint compartments (**b**) leg sides (**c**) and cartilage layer (**d**) on mean values and standard deviation of T2* relaxation times in ms

Analysis of the T2* relaxation times demonstrates different values depending on the defined region of interests of the knee (Fig. 2). The superficial region showed increased T2* values compared to the deeper region (*p* < 0.001) (Fig. 3d). A significant increase of relaxation times of the femoral cartilage could be detected from anterior to intermediate to posterior (*p* < 0.001) (Fig. 3a). The overall highest T2* values were detected in the superficial, posterior femoral zone (42 ± 1.7 ms) (Figs. 2 and 3a). There were no significant differences between the right and left leg as well as the medial and lateral compartment (Fig. 3b and c).

### Leg axis analysis

Analysis of the leg axis geometry revealed a mean static alignment of 6.6° ± 2.5 varus and a mean dynamic alignment of 5.1° ± 2.6 varus.

### Correlation analysis of T2* Mapping and leg axis analysis

**Fig. 4:**
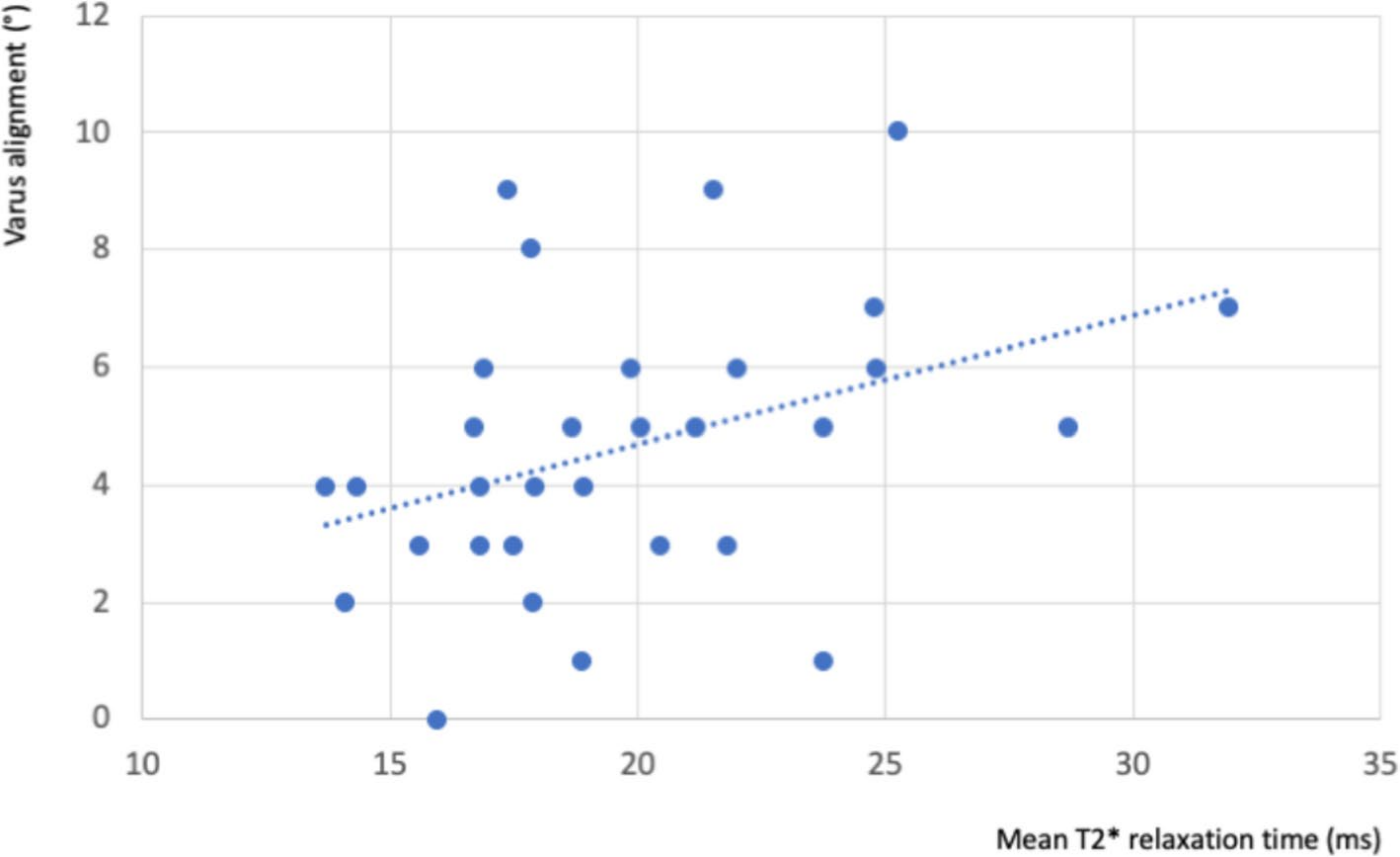
Correlation analysis of T2* relaxation times in ms of the femoral, posterior, deep region of the medial knee compartment and leg axis alignment in degrees (°)

Correlation analysis of leg axis and MRI T2* mapping revealed a significant correlation for varus alignment and T2* values of the femoral, posterior, deep region of the medial knee compartment (*r* = 0.4; *p* = 0.03) (Fig. 4). Subgroup analysis of players with static varus alignment > 6° (*n* = 10, 20 legs) demonstrated a significant increase in T2* values in the femoral, posterior, deep region of the medial compartment compared to players with a less pronounced leg axis deviation (*p* = 0.003).

## Discussion

The most important finding of this study is the correlation between varus alignment and T2* values of the femoral, posterior, deep region of the medial knee compartment. This study demonstrates the successful use of T2* MRI mapping, radiation free leg axis analysis via DIERS formetric 4D and their combination in order to gain important information about the interaction of leg axis and early cartilage alterations in professional youth soccer players. Furthermore, this study validates the typical distribution of T2* relaxation times with an increase from deep to superficial as well as the frequent occurrence of genu varum.

Increased T2* relaxation times of the knee cartilage are considered to reflect early changes in the development of cartilage lesions as well as osteoarthritis [22–24]. Several studies could identify a different distribution of relaxation times within the knee cartilage. Typically, the cartilage shows increased T2* values in the superficial layer [16–18, 24]. The results of our study in a cohort of young professional soccer players validate these results, showing increased T2* values in the superficial layer. Our results furthermore demonstrate increasing values from anterior to posterior with the superficial, posterior femoral zones to yield the highest T2* values. This is comparable with recent data of Schenk et al. examining a similar study cohort [16]. In contrast, data of Zhao et al. and Zhu et al. showed the highest values in anterior to central regions but the patient collectives did not specify for professional athletes [25, 26]. The posterior located increase in T2* values might be due to a load shift to the posterior femoral condyles during knee flexion and therefore pronounced in running related sports such as soccer where posterior parts are subjected to greater stress [17, 27, 28].

In this study a mean static leg alignment of 6.6° ± 2.5° varus could be detected and is therefore in accordance with previous data stating that professional soccer players demonstrate an increased prevalence of genu varum. Still, a direct comparison of the absolute numbers is not possible since different measurements (intercondylar/intermalleolar distance, hip-knee-angle, medial proximal tibia angel, lateral distal femoral angle, Q-angle) were used to define varus deformity [5, 29–32]. Varus alignment induces a mechanical load shift towards the medial joint compartment. This pathological load shift increases the risk of degenerative cartilage changes up to manifest osteoarthritis [12, 33–37]. Several studies highlight the necessity of an additional dynamic evaluation of the leg axis since dynamic measurements might differ from a solely static view [12, 38–40]. In this context our data reveal the presence of dynamic varus alignment with a mean of 5.1° ± 2.6° in young professional soccer players. This could be a contributing factor in altered knee joint loading with an increased knee adduction moment, which cannot be solely explained by the static mechanical leg axis [41]. Still it needs to be considered, that the results of this study represent a two-dimensional view from posterior where potential hip joint rotations during dynamic conditions are not recorded and therefore might alter the true mechanical leg axis. However, Ossendorf et al. recently demonstrated a strong correlation for static as well as dynamic measurements to the long-leg-axis radiography which is considered to be the current gold standard in assessment of the leg axis [15].

This study demonstrates the relation between T2* relaxation times and leg axis alignment.

Schenk et al. hypothesize that alterations of the cartilage structure begin superficially near the articular surface and will proceed to the deep layer in a more advanced stage of overuse -induced degenerative changes. In contrast, our findings might indicate that under the influence of varus related medial joint loads, the deep cartilage layer alters first. Taking these results into consideration in the developmental pathway of osteoarthritis, our observations could be a part of early changes within the osteochondral unit which is known to play an important role in the early development and progress of osteoarthritis [42, 43]. Still, the observed changes might also represent physiological adaptions due to increased joint loads in a cohort of professional youth soccer players.

Recently, a more detailed mapping of the knee cartilage dependent on T2* values was suggested to identify regions at risk. The medial cartilage regions, especially the medial posterior zones, were described to be vulnerable and at extra risk in young professional soccer players [16]. In this context the results of this study demonstrate the impact of varus alignment on these regions. In our study, soccer players with a varus deformity > 6° showed a significant increase in T2* relaxation in the femoral, posterior, deep region of the medial knee compartment. Also, this specific region showed a significant correlation of T2* relaxation time and the degree of genu varum. Clinical data prove these regions to be frequent locations of manifest cartilage lesions underlining the clinical relevance [1]. Interestingly, the overall results of our study did not find a significant difference between the medial and lateral compartment. Therefore, the correlation analysis of leg axis geometry and T2* relaxation times indicate the crucial role that varus alignment might play especially in the early development of these lesions and before they become clinically noticeable. Therefore, there should be a special focus on sports-related varus alignment in the development of preventive programs since it might significantly influence the development of cartilage injury and osteoarthritis in the long run. Our study demonstrates the combined use of radiation free leg axis analysis and T2* mapping to be a feasible way to gather important information on regions at risk of cartilage injury in a high-risk patient collective.

Limitations of the study include the relatively low number of examined soccer players, the possible inaccuracy within the different measurements of the DIERS leg axis analysis and that the measurements were not taken by an independent observer. Still, all measurements were carried out on the same day by one experienced sports medicine physician specializing in movement analysis. A control group of soccer players with a strictly straight leg axis would further strengthen our results. Still the cohort of examined soccer players already demonstrated different degrees of leg axis alignment allowing to interpret the impact of varus deformity and answer the hypothesis of the study. All T2* measurements in this study need to be considered with care since the pathological predication of elevated T2* values still is not completely understood and histological proof has not been performed.

In conclusion our study demonstrates that varus alignment in young soccer players is associated with MRI T2* changes in the deep cartilage layer of the medial, posterior, femoral compartment and might therefore be a contributing factor in the early development of manifest cartilage lesions. Still, validation in a bigger study cohort and especially a longitudinal examination of this lesions to confirm the pathological pathway are mandatory.

## Data Availability

The datasets used and/or analyzed during the current study available from the corresponding author on reasonable request.
